# 12 tips to hear the voices of introverts in medical education … and to improve the learning climate for everyone

**DOI:** 10.15694/mep.2021.000107.2

**Published:** 2021-09-03

**Authors:** Reinoud de Jongh, Anne de la Croix

**Affiliations:** 1 Erasmus University Medical Center; 2 Erasmus University Medical Center; 3 Amsterdam UMC; 4 Amsterdam UMC

**Keywords:** inclusivity, educational strategies, curriculum design, assessment, classroom management, education, teachers, students, faculty development, reflection

## Abstract

This article was migrated. The article was marked as recommended.

Small group, highly interactive teaching is growing in popularity, making medical school stacked in favor of the extraverted student. The resulting discomfort experienced by introverted students is well documented and troubling. Not just for their wellbeing, but also for the educational climate as a whole. Everyone misses out on learning opportunities when a group of students is not heard or feels uncomfortable to speak up.

In this piece, we offer twelve tips to create a safe and comfortable learning environment for all students, regardless of where they find themselves on the introvert-extravert continuum. In these tips we will focus on self-knowledge and perceptions of silence, didactic strategies and learning activities, starting a conversation to become more aware of differences and reflect on them, training for introverted students and fair assessment.

## Introduction

Small group teaching in medical education is increasingly popular; there has been a ‘shift toward more interactive teaching strategies ... and group learning venues (e.g., study table in pods, more open design libraries) ( Davidson, Gillies and Pelletier, 2015). In these teaching sessions, active participation is valued, meaning that students are expected to speak up. An unintended consequence of this preference for talk is that medical school has an extrovert ideal: ‘Medical school seems stacked in favor of the extrovert, ... with group activities, rapid fire questions, and all that talking.’ ( Riddle, 2014) This leaves introverts feeling ‘like misfits, being afraid of being wrong or misunderstood ... , feeling a need to change their identities to succeed in medical school’ ( Davidson, Gillies and Pelletier, 2015). Introverted medical students experience a myriad of difficulties during their clinical training, too. They experience more stress and anxiety, struggle to give off a good first impression in the fast-paced scheme of clinical rotations, and get lower evaluations because physicians in the clinical phase mistakenly interpret their behavior as lacking interest or enthusiasm ( Davidson, Gillies and Pelletier, 2015; Fleming, 2015; Noureddine and Medina, 2018).

Without delving deeply into the origins of the terms ‘introverted’ and ‘extraverted’, we will highlight the basic characteristics. Extraverts get energy from interaction with others and like to share ideas with others to help develop their thinking, whereas introverts need to recharge on their own after much social contact and prefer to share ideas only when they are fully formed. For group discussions, the important distinction is that extraverts ‘talk to think’ and introverts ‘think to talk’ ( Lee, 2017). One can imagine that medical school is a very different place for introverted and extraverted students, as illustrated by this introverted student’s quote: ‘Group learning has been a fixture of our curriculum since day one, as has the fabled tradition of being put on the spot and quizzed by teachers in front of peers. Networking is still the preferred method for finding research opportunities. And the famous learning philosophy of “see one, do one, teach one” has been jarring for me as somebody who likes to take time for deliberation and reflection - a bit like being pushed out of an airplane at 10,000 feet.’ ( Fleming, 2015)

This discomfort experienced by introverted students is troubling. Not just for their wellbeing, but also for the educational climate as a whole. Everyone misses out on learning opportunities when a group of students is not heard or feels uncomfortable to speak up. It is important for all students to create a safe environment that embraces diversity, only then will all viewpoints be heard. Luckily, there are ways to influence this learning climate and use techniques to stimulate a truly inclusive discussion, in which introverted students’ are also heard.

In this publication, we (an introvert and an extravert with ample small group teaching experience) would like to share our twelve tips to help teachers and tutors cater for both introverts *and* extraverts, without forcing people to be anything else than their authentic selves. Tips 1 and 2 are considerations for educators, as some introspection and reflection is needed to become aware of introvert-extravert differences. This reflection can help implement the other tips successfully. Tips 3, 4, 5 and 6 focus on teaching techniques and curriculum design. Tips 7, 8, 9, and 10 are ways of making students aware of differences and using these to their advantage. Tips 11 and 12, finally are about assessment and evaluation.

## Tip 1: Teacher, know thyself!

Our first tip is to consider where you, the educational professional, are positioned on the introversion-extraversion continuum. After all, this might influence your idea of ‘a good teaching session’, and how you interpret and evaluate student behaviour.

If you are a more extroverted teacher, you might lean towards teaching strategies such as brainstorms, debates, and group discussion. ‘An extroverted classroom can be a lively and ener-getic public forum ... . It is fast paced, changing direction frequently, with distractions, noise and high stimulation. Marks, favours and other rewards are given for extroverted participation and those who don’t participate the way that extroverts do can be neglected or singled out.’ ( Thompson, 2012) However, if you are an introverted teacher, the picture sketched in the previous quote might sound quite exhausting. You might focus on preparation, written work, and well thought-through arguments.

Additionally, knowing your personality type, you could stop and wonder whether or not you hold any potential implicit biases against introverted students, who may appear either unmotivated, unprepared or both, or against extroverts, who may appear to be “arrogant, bossy, or lacking in listening skills” ( Colley, 2018).

We advise teachers to read about the introversion-extraversion scale. A good start is the book ‘Quiet’ by Susan Cain ( Cain, 2013), which contains a test to get a rough idea of your own position on the scale (see https://www.quietrev.com/the-introvert-test/). Alternatively, more elaborate Big Five personality assessments can be found online.

## Tip 2: Become ‘silent literate’

Teachers often experience silence as uncomfortable: their own silences feel ‘marked’ and longer than they actually are ( Jaworski and Sachdev, 1998; Star and Strickland, 2008). Additionally, speech is often interpreted as a sign of participation, whereas silent students are seen as unmotivated and disengaged ( Bean and Peterson, 1998; Jaworski and Sachdev, 2004; Schultz, 2009; Jin, 2014). The necessity of silence in the learning process is often overlooked, as are the thresholds for students to take the floor and talk in the classroom ( Jackson, 2002; Cain, 2013; Ryu, 2013; Frambach *et al.*, 2014). One potential threshold could be introversion, but cultural background is another one. An effect of the importance of hierarchical relations in Middle Eastern and Asian students, for example, can be that students feel the tutor should not be challenged, as openly disagreeing with a teacher is considered disrespectful. As another example, a strong focus on group relations and saving face can result in students only speaking up if they are sure that they are correct, and being hesitant to ask questions as it shows a lack of knowledge ( Frambach *et al.*, 2014). In other words: silence does not equal disinterest.

Silence can also result from a lack of psychological safety, defined as ‘a climate in which people are comfortable expressing and being themselves’ ( Edmondson, 2018). As Edmondson (2018) points out: ‘Psychological safety exists when people feel their workplace is an environment where they can speak up, offer ideas, and ask questions without fear of being punished or embarrassed’. While psychological safety is important for all students, this is especially so for introverts. In the words of Riddle (2014): ‘We introverts are inclined to hold back, answering only when we’re 110% sure we know the answer’. See tip 9 for some advice on how to enhance psychological safety, which applies to teachers and students alike.

To do right to all students, educational professionals could do with more insight into the reasons for silence, but also in the power, effects, and possible uses of silence in the classroom. We advise to learn about different functions of silence, so you can recognize them better and use them as a teaching tool. Silence can function as a way to relax and focus, to guide attention, to let people think and reflect, to allow for emotions in the classroom, and to use more senses in the learning process. The term silent literacy is appropriate here: ‘the ability to employ and understand silence appropriately in context’ ( Bao, 2014).

So how can we use silence? As we suggest in this paper, becoming silent literate is a process that includes self-knowledge, becoming comfortable with silence, using silence in the classroom when giving tasks, and asking questions about their experiences with silence. Practically, it can start with teaching tricks as presented in the next tips.

## Tip 3: Provide advance notice

Give students time to prepare for classroom activities, and let them know which topics will be discussed. Introverted students need more time to let ideas form, and prefer to think or analyze matters before talking. Knowing in advance what will be teaching material, is useful for introverted students ( Davidson, Gillies and Pelletier, 2015; Colley, 2018).

Ways of doing this could be to give students a preparatory assignment before class, especially when it concerns a brainstorm, a debate, or discussion. This can be done as homework, but mentioning the agenda of the teaching session at the start of the class can also help. And on an even smaller scale, just mentioning a question and saying ‘no need to answer now, but I will get back to you later’ can give introverted students a better chance to be prepared for group interaction. Another good classroom activity is to let students think in silence or write some thoughts out on paper, before entering a group discussion. Finally, be clear about the roles people will play in discussions. Structure discussions, and be clear about expectations, as this is a way to invite everyone into the discussion ( Cain and Angelo, 2019).

## Tip 4: Pause after asking a question

When you ask a group of students a question, give them some time to think before gathering answers. If there is no thinking time, it is almost guaranteed that the first people to speak up, are the extraverted students. This way, both teacher and students may miss out on the reflective insights of introverted students, and instead, discussions are dominated by the insights of extraverted students, which tend to be more spontaneous and improvised, but therefore also sometimes lacking in depth and reflection ( Colley, 2018).

Classic research has shown that a waiting time of 3 seconds after asking a question can lead to more responses, a better explanation of the teacher, and longer, better, and more appropriate answers from students ( Rowe, 1974). A more recent review on waiting time (less than 5 seconds) confirms these findings: there are positive learning outcomes that are amplified by slowing down the tempo of instruction ( Tincani and De Mers, 2016). So, in order to enrich discussions, offer some thinking time. An added advantage is that students can practice with being quiet, observant and reflective before responding, a skill that can be very useful for physicians, for example in bad news consultations ( Lee, 2017).

We would advise to explicitly introduce this thinking time, so introverts can think on their own, and extraverts can analyse and possibly modify their initial response, potentially leading to deeper insights. Note that this thinking time does not necessarily mean ‘doing nothing’ - teachers and tutors can encourage students to draw, doodle, make notes, or write a ‘one minute paper’. This way, both introverts and extroverts have to make concessions, but also benefit: introverted students are allowed time to reflect, but are expected to share with the class. Extroverts can still contribute, but are expected to reflect first ( Colley, 2018).

An important thing to consider is that tutors might feel uncomfortable with silence, especially when they are extraverts themselves ( Edmunds and Brown, 2010). In this case, it helps to set a timer for the silent thinking time, and mention that the silence may be uncomfortable for some, but will help the discussion and the consequent learning. The extraverted author of this paper (AC) has learnt a lot about her own intolerance and discomfort around silence by doing this.

## Tip 5: Ask for contributions in different ways

When initiating a group discussion, try different ways of gathering viewpoints, questions, or ideas.

As an introverted student noted in one of our workshops: ‘When everybody immediately starts shouting out their answers, I tend to think: “never mind”’. Simply posing a question in a group therefore tends to elicit responses only from the more extraverted students.

There are several teaching strategies you can use to ensure that all students contribute, even if they might not do so verbally. See Table 1 for some teaching tips. In all these strategies, time is granted to introverts to think before having to speak, as Susan Cain notes: ‘students who might not have shared their ideas if asked coldly, are now more likely to do it, because they’ve had the opportunity to articulate them just to one other student first’ ( Cain and Angelo, 2019).

The switch to online education due to Covid-19 may have been a blessing in disguise in this regard. Asking students to use the chat function to contribute to a discussion also gives introverted students time to formulate their answer, while the virtual ‘hand-raise button’ makes it easier for them to contribute once they have decided to do so, without having to ‘elbow their way into’ the discussion.

**Table 1:** How to ask for contributions in different ways

**post-its.** Step 1: After you pose your question, let students think individually and write down their answers on one or several post-its. Step 2: You ask the students to cluster the post-its, for example on a white board or on a table. Step 3: you discuss the similarities and differences, or answers that stand out. In an online environment, you can use the program Padlet ( https://nl.padlet.com/).
**think-pair-share (TPS)**. Step 1: After asking a question, allow students to think individually for 2 minutes. Step 2: students pair up with another student and discuss for 4 minutes. Step 3: students re-group and some or all of the pairs share their ideas with the rest of the class.
**use software:** in programs such as Mentimeter, you can pose a question and ask students to think about it and then respond on their smartphone or laptop. The answers will be visible live on the computer screen, for example as a word cloud or a flowing grid. You can then ask students to explain their answers or to elaborate.

## Tip 6: Offer variation in your curriculum

Variation in the teaching schedule will lead to the inclusion and participation of more students. Some teaching contexts show a good fit with introversion, such as a didactic lecture, where students are listening and taking notes, while others show a strong fit with extraversion, such as a discussion group, or bedside hospital rounds.

When there is a poor fit, introverted students will seek to recharge themselves by seeking out a more quiet, subdued situation, whereas extraverted students will try to recharge by seeking social stimulation ( Davidson *et al.* 2015). An extraverted student in our workshop remarked that a 45-minute lecture under-stimulated her, causing her to lose focus. What she really wanted to do was to be active, and talk about the topic. In the think-pair-share strategy (tip 5), some extraverted students wanted to skip the thinking phase, as they knew immediately what they wanted to say and didn’t see any point in being quiet for 2 minutes, often finding it annoying and sometimes even repulsive. A day with three very interactive teaching sessions is exhausting for introverts, whereas a full day of listening to lecturers is exhausting for extraverts.

A solution is to be conscious of the demands of the type of teaching, to keep an eye on the students’ daily and weekly class schedule, and to potentially replan it to allow both extraverted and introverted students to recharge themselves. Alternatively, as discussed in previous tips, making small adjustments to your teaching strategy within the classroom setting can help too

## Tip 7: Start a conversation about who talks and when

Talk to students about differences in the way they communicate. This might help group dynamics in classroom settings, but also teaches students about possible interaction patterns that can occur with colleagues in the future, or in their conversations with patients.

A way to make students aware of their communicative behavior, is to shake up the normal flow of conversation, for example with a **‘paperclip discussion’**. For this technique you need a topic for discussion, and you form groups of 4 or 5 students. Each student gets three paperclips. Whenever a student wants to contribute to the discussion, they have to place one of their paperclips on the table. This starts a timer for 30 seconds of (uninterrupted) speech time. When students are out of paperclips, they can no longer contribute to the discussion. The teacher observes the process: who is the first to put down a paperclip? Who runs out of paperclips first? Ask students (introverts first!) about their experiences afterwards. We spoke to an extraverted student who remarked that the exercise taught her “to make a conscious choice of what I wanted to say and when was the right time to do so”. Another extraverted student said: “It made me realize that time is valuable”. Several students remarked that they would try to give introverted students more space in future discussions, and not always be the first to answer.

A different technique with a similar goal is to use a ‘talking stone’ (only the person holding it can talk). Alternatively, the teacher could simply count the number of times people take the floor, and reflect on this with students: did they notice who spoke and how much? Let them discuss in groups why they did or did not speak up, and how they feel about the division of speaking time. We have experienced eye-opening moments in groups that suddenly became aware how the floor time was actually divided.

## Tip 8: Talk about differences

Talk about differences, perhaps in the context of teaching around communication or professionalism. This can be a very valuable and eye-opening exercise.

In our introvert-extravert workshop, we divided groups into extraverts and introverts based on a brief personality test. We then interview each group and ask them about their strengths, weaknesses, annoyances, and misconceptions other people have about them.

Extraverts list taking the lead, being spontaneous and enthusiastic, and speeding up discussions as their strengths, for example, but also mention that they would sometimes like to think first before they speak up, and would like to be better at being on their own. Introverts mention their focus, being modest and mindful, and being able to form in-depth connections as their strengths, but say they sometimes think and worry too much, and would like to be less reserved, interact more, and contribute more easily to a discussion. Talking about misconceptions also proved to be enlightening: introverts mention for example that people think they are shy, boring, have a dull life and do not like to collaborate. Extraverts, on the other hand, mention people thinking they are superficial, and never insecure or shy.

The annoyances extraverts have about introverts can be briefly summarized as: ‘say something!’. Vice versa, introverts feel annoyed about extraverts’ ‘blah blah blah’ and leaving no room for others.

Talking about these differences seemed to promote understanding, mutual respect, and the willingness to take these differences into account. At the end of one of our workshops, when asked about their eye-openers, a student remarked: “That introverts are just as motivated to contribute to a discussion, but they need more thinking time to do so”. Another student said: “I will always be tempted to be the first to answer, but I’m more conscious now of how this can be ‘too much’, and I should take other students into account”.

## Tip 9: Teach extraverted and introverted students how to collaborate

When working in collaborative groups and teams, extraversion and introversion both have advantages and disadvantages. An awareness of differences in personality on can also be used to promote collaboration, which is one of the CanMEDS competencies that every physician needs to acquire ( Frank and Danoff, 2007).

In situations where proactive suggestions are important, extraverted leaders should adopt a more reserved, quiet style, something which comes naturally to introverts ( Grant, Gino and Hofmann, 2011). Introverted leaders, on the other hand, can enhance team performance by actively encouraging proactive behaviors when employee proactivity is low. Extraverts may benefit from active listening and learning to use their talkative, outgoing nature to facilitate as opposed to dominate a group discussion ( Cullen-Lester *et al.*, 2016). Introverts, on the other hand, can be trained to develop more energy in their interactions, something which comes naturally to extraverts, for example by focusing the conversation on possibilities and making progress.

When it comes to collaboration, extroverts and introverts have a lot to learn from each other, and a focus on the role of personality in team functioning can improve collaboration ( Clinebell and Stecher, 2003). A student-team intervention in which the personality of the team members was assessed to enhance the team development process, was evaluated by students as helpful and enhanced their understanding of their own behavior, as well as that of their team members. Students used their awareness of differences to promote team functioning, for example: *"... because [student] was an introvert and we focused on getting his opinion."* ( Clinebell and Stecher, 2003)

Collaborative skills, for example waiting patiently when a team member is thinking and praising and thanking others for their contributions, can also help introverted students to feel more comfortable when learning in groups ( Jacobs, 2014). This also includes enhancing psychological safety within the team or classroom, for example by training active listening skills (such as summarizing and asking (open) follow-up questions to understand the other person’s perspective, while refraining from giving your opinion) and teaching students to adhere to the rules of effective feedback.

At the end of our introvert-extravert workshop, participants often remarked on the role of personality in collaboration: “We need each other. Both personalities have their strengths and weaknesses, and we should learn to recognize these in each other and use them”. Another student mentioned: “Being willing to learn from each other, so that you understand each other better and can choose the right strategy”.

## Tip 10: Organize training sessions for introverted students

Organize training sessions and/or peer learning groups for introverted students, in which they can learn how to balance their own style while coping with demands and pressures of medical school and/or clinical practice. Note that this should be an open-access course, welcoming any student who is interested, and should not be mandatory.

First and foremost, this means teaching introverted students how to play to their strengths, after all, introverts are known to be thoughtful, analytical, and adept listeners, which often can be “a strength rather than a weakness” in clinical practice ( Gillett, 2016). For example: seeking out one-on-one time with their patients where they can take advantage of their listening skills ( Riddle, 2014; Fleming, 2017), or using their ability as a quiet people watcher to observe team dynamics, in order to integrate well into the team ( Riddle, 2014).

Second, introverts can share tips and tricks on how to cope better in an ‘extraverted training context’. One example mentioned by an introverted (former) medical student: prepare and practice ( Riddle, 2014), such as memorizing the first line of a presentation, or writing out what you want to report on rounds. Another tip: sharing your hard work and accomplishments with your team ( Fleming, 2017). One of our own students found this advice to be really helpful in getting noticed and appreciated, but, being an introvert, it had simply never occurred to her to do so. Other examples include speaking up, even if you are hesitant about the answer ( Riddle, 2014; Fleming, 2017), and recharge, by not going out on your one night off, but instead staying in and reading a book or watching Netflix with a friend. During the day, students can also try to seek out ‘restorative niches’ for example by having lunch by themselves ( Little, 2010; Riddle, 2014).

Third, introverted students could learn to occasionally act in a (pseudo) extraverted manner to advance personal projects, but should realize that protractedly acting out of character can lead to potential burnout ( Little, 2010). The introverted author of this paper (RJ) loves to lecture to large student audiences, for example, and has come to regard this a ‘performance’, but always makes sure he has some alone time afterwards in order to recharge.

## Tip 11: Judge participation in different settings

The strategies mentioned in tip 5, think-pair-share (TPS), post-its and programs such as Mentimeter, also provide different ways to assess students. In TPS, you can walk around and observe the discussions in pairs and small groups. You might find that students that would never speak up in a large group discussion, turn out to be very active when discussing in pairs.

And even if introverted students do not speak in the group interaction that follows these teaching strategies, their ideas are still included in the discussion, as they are visible to all in the post-its or Mentimeter. Once visible, you can ask the student to elaborate on their input. This broadens the scope of ways in which to assess students and get a glimpse of their development as future health care professionals.

## Tip 12: Include ‘introverted skills’ in assessment

Change assessment criteria so attention is also given to more ‘introverted skills’. Evaluation forms in small group education and clerkships typically include items such as: ‘participates in session’ and ‘willing to initiate discussion’ ( Davidson, Gillies and Pelletier, 2015). This puts introverted students, with their more reflective thinking style, at a disadvantage, especially since by ‘participation’ teachers generally mean: ‘talking’.

Active listening is also a vital part of participation ( Spencer, 2015), and Cain even recommends replacing the term ‘classroom participation’ with ‘classroom engagement’ ( Cain and Angelo, 2019). Other suggestions are to add items such as: ‘thinks before speaks’, ‘listens to peers before engaging’ and ‘offers a synthesis of the information’ ( Davidson, Gillies and Pelletier, 2015). An added benefit of this approach, is that extraverted students are encouraged to develop these skills. A useful tool to get inspired is the so called ‘Quiet student engagement rubric’ developed by The Quiet Revolution group https://www.quietrev.com/wp-content/uploads/2018/11/RS-Quiet-Student-Engagement-Rubric.pdf.

## Conclusions

While more and more teaching happens in small groups, using discussions and interaction, we need to offer teachers enough strategies to include all students in these discussion. Introverted students are often at a disadvantage in ‘spontaneous’ verbal tasks such as brainstorms or free flowing discussions. We feel that this is unfair and a missed opportunity for both introverts and extraverts. We invite teachers to investigate their own place on the introversion-extraversion scale, and reflect on their ideas and feelings around silence. By making small adjustments to teaching and curriculum design, you can involve more students and even teach them about their differences. After giving many workshops about this, we have learnt that both introverted and extraverted people will not regard group interaction in the same way after learning more about how the interactional floor is taken, given, and shared.

## Take Home Messages

• Teaching strategies and assessments tend to be biased towards extraverted students.
• Both teachers and students should reflect on their position on the introvert-extravert scale.
• Talking about and learning from differences in personality promotes inclusion and collaboration.
• Teachers should know different ways of asking students to contribute to group discussions.
• ‘Introverted skills’ need to be recognized, trained and rewarded in education.

## Notes On Contributors

**Reinoud de Jongh** is an introvert. He is a lecturer at the Department of Psychiatry, Section Medical Psychology, Erasmus Medical Centre, The Netherlands. Interestingly, his work as teacher asks a lot of ‘extroverted skills’.

**Anne de la Croix** is an extrovert. She is an assistant professor at Amsterdam UMC, Faculty of Medicine Vrije Universiteit Amsterdam, Research in Education, Amsterdam, The Netherlands. Interestingly, her work as researcher asks a lot of ‘introverted skills’.

Together, they have developed a workshop called ‘The talkative student and the wallflower: how to cater for both extravert and introvert students in the classroom’. They delivered the workshop at a variety of events.
