# Supplementary material for: 12 tips to hear the voices of introverts in medical education … and to improve the learning climate for everyone
Source: MedEdPublish (2016). 2021 Sep 3;10:107. Originally published 2021 Apr 30. [Version 2] doi: 10.15694/mep.2021.000107.2 (PMC10939523; doi:10.15694/mep.2021.000107.2)
Supplement: Peer review history

# Reviewer report: Reviewer response for version 2

**DOI:** 10.21956/mep.20217.r31304

**Published:** 2021-10-07

**Authors:** Dimitri Parra

**Affiliations:** 1 The Hospital for Sick Children

This review has been migrated. The reviewer awarded 5 stars out of 5 The authors present a paper in which they describe 12 tips to facilitate the teaching of introverts, and at the same time of the extroverts.I think it is an excellent paper and I really enjoyed reading it.It is crucial the fact that it is mentioned the need of the teachers to characterize in which position of the spectrum they are. This is important as it requires an exercise of self-reflection. All the tips presented are very valuable. As Prof Jennings mentions in her review, this article should be shared widely. Congratulations for the publication and I hope you continue doing research in this area. An interesting area to explore will be clinical training experience advice for introvert and extrovert and how to adapt current communication skills training to them. Also, integration to interdisciplinary and interprofessional activities. Health care professionals are frequently speaking in public and preparing presentations (now many in TV due to the pandemic) and I believe we do not have a clear training how to train the different personality and communication styles to adapt and integrate to this environment.

Reviewer Expertise:

NA

No decision status is available

# Reviewer report: Reviewer response for version 2

**DOI:** 10.21956/mep.20217.r31305

**Published:** 2021-09-10

**Authors:** Barbara Jennings

**Affiliations:** 1 Norwich Medical School

This review has been migrated. The reviewer awarded 5 stars out of 5 Thank you to the authors for the additional time spent revising their article in response to the reviewers’ comments linked to version 1. The grouping of tips around aspects of teaching/curriculum, reflection and assessment practice creates a very helpful and logical structure. I do think this excellent article should be shared widely - and am sure it is already being read by PBL facilitators and folk interested in group dynamics and learner well-being. The focus on psychological safety is so important and timely. Perfect reading for those about to join small group forums this autumn.

Reviewer Expertise:

NA

No decision status is available

# Reviewer report: Reviewer response for version 2

**DOI:** 10.21956/mep.20217.r31306

**Published:** 2021-09-07

**Authors:** P Ravi Shankar

**Affiliations:** 1 American International Medical University

This review has been migrated. The reviewer awarded 5 stars out of 5 I enjoyed reading this article. I have facilitated small group learning sessions and workshops on different continents. I was aware of introverts and extroverts but had not really thought about the impact on small-group learning. Small group learning is becoming commoner globally and recently due to pandemics, these are being facilitated online. As the authors mention sessions online do offer some advantages to introverts. They are more likely to pen down their thoughts and ask questions in the chat. Online sessions also have problems in that you mainly see only the face of the participants and may not be able to notice the body language. Participants may not switch on their cameras, and some may have unstable internet connections. The article offers useful advice to a diverse group of educators.

Reviewer Expertise:

NA

No decision status is available

# Reviewer report: Reviewer response for version 1

**DOI:** 10.21956/mep.19020.r27302

**Published:** 2021-07-30

**Authors:** Barbara Jennings

**Affiliations:** 1 Norwich Medical School

This review has been migrated. The reviewer awarded 4 stars out of 5 As a PBL facilitator for undergraduate medical students, I have tried to address the introvert-extrovert spectrum with the groups with whom I work - and found I myself saying “hear-hear” as I read the tips suggested in this interesting and useful article. I think it will be read by PBL facilitators and anyone interested in group dynamics and the wellbeing of undergraduate students. I will certainly be sharing the article with undergraduate students and in faculty-development sessions. The take home messages and the notes on contributors section added to the usefulness of the article. The authors introduce us to the particular problems that introverts face in interactive, small group teaching. The raise the challenge within dynamic groups of the dichotomy between those who ‘talk to think’ and others who ‘think to talk’ – and of course tutors as well as learners are group members (see Tip 1).The pandemic and use of remote teaching has made me develop methods to “ask for contributions in different ways” and the authors list great ideas for this (Tip 5).Suggestions for a version 2:• Limited background literature was included in the introduction - though this may reflect that this is an under-studied area of curriculum design and delivery?• Could literature exploring intersectional factors (in addition to the personality) that impact learners, and their psychological safety, be complementary to the introduction or any of the tips?• The use of different parentheses and gaps in quotations is a bit odd.

Reviewer Expertise:

NA

No decision status is available

# Reviewer report: Reviewer response for version 1

**DOI:** 10.21956/mep.19020.r27304

**Published:** 2021-05-12

**Authors:** Mary Higgins

**Affiliations:** 1 University College Dublin, National Maternity Hospital

This review has been migrated. The reviewer awarded 4 stars out of 5 I loved this paper and found it a very practical piece with hints that I can begin to use as soon as possible. My only small comment is that in your quotes in the introduction that the use of brackets as well as dots (...) to break up a paragraph or quote meant that I thought you had forgotten some references. I haven't seen this before and think with this referencing style using the dots alone would be more appropriate.

Reviewer Expertise:

NA

No decision status is available

# Reviewer report: Reviewer response for version 1

**DOI:** 10.21956/mep.19020.r27305

**Published:** 2021-05-02

**Authors:** Rajaei Sharma

**Affiliations:** 1 College of Medicine and Health, University of Exeter

This review has been migrated. The reviewer awarded 4 stars out of 5 This is a very interesting and necessary area of discussion given the ongoing development of medical education; the increasing prevalence of small-group teaching alongside the increasing numbers of medical students. The abstract is concise and suitable. It may be additive to list the tips or highlight the best here. The introduction offers a good level of background but it may be useful to have a more literature references/discussion as to any similar work; especially if there are any other 12 tips! Furthermore, the authors themselves do not state the intended audience until the 1st tip which may be a useful discussion point. Tips 1/2/6 are a little lacking in solid recommendations – they are more considerations! For example, tip 1 could include specific ideas about what a teacher should consider once they have identified as extro/introverted. The open source recommendations are great for the readership. Tip 7 is a great comparison – it provides concrete new ideas and suggestions.Tips 3-5 read well and are very pertinent.Tip 10 is interesting and could be controversial. Identifying a certain group of students based on personality and providing targeted teaching requires a careful approach. This type of teaching should be open-access and broad in scope. There should be some attention paid to this in the text. Tips 11 and 12 feels a little lacklustre! Tip 12 is useful but suddenly introduces the aspect of assessment which in very different to the general theme of the tips. The conclusion and take home messages are well written and summarise the piece well. Overall, this is a well-balanced piece that will likely be additive and informative to medical educators worldwide. The points raised here are all additive in nature and with revisions this would be a great piece to read.

Reviewer Expertise:

NA

No decision status is available

# Reviewer report: Reviewer response for version 1

**DOI:** 10.21956/mep.19020.r27303

**Published:** 2021-05-01

**Authors:** Virginia Randall

**Affiliations:** 1 Uniformed Services University of the Health Sciences

This review has been migrated. The reviewer awarded 5 stars out of 5 I learned a lot from this paper. The 12 tips bring the topic into the teaching milieu in a way that will be easy to implement. I also learned about introvert/extrovert persons and their behavior. I have always been shy, I considered it a character flaw, but while reading this paper I began to see it as a strength to be able to add well thought out comments to group discussions. The authors warning about implicit bias against introverts is important and I think happens all too often. Many of the tips can also apply to group discussions which include students of color, who may hang back from expressing their opinion for fear of disapproval.

Reviewer Expertise:

NA

No decision status is available

# Reviewer report: Reviewer response for version 1

**DOI:** 10.21956/mep.19020.r27301

**Published:** 2021-04-30

**Authors:** Subha Ramani

**Affiliations:** 1 Harvard Medical School, Brigham and Women's Hospital

This review has been migrated. The reviewer awarded 3 stars out of 5 I really enjoyed reading this manuscript and agree that this is a topic that needs to be addressed particularly in the current educational environment. With increasing use of virtual teaching and learning, the gap between extroverts and introverts widens even more.Introduction- I agree with the points raised in the introduction, was able to reflect on the definitions of introverts and extroverts. I do agree with the implication on well being as well. A couple of additional points I would have liked to have seen are: how these factors play out in the virtual educational setting and perhaps use the terms implicit bias against introverts and the importance of psychological safety in inviting the introverts into the conversation.While I agree with and learned from much of the content under each tip, It seems some of the tips could have stronger and more powerful headings.Tip 1- Knowing oneself is indeed important, but I believe that teachers need to step up further and know and acknowledge their biases towards/against learners different from themselves. Knowing oneself is only the first step.Tip 2- This title could be stronger. The content is much more than becoming the silent literature as there are strong implications of cultural backgrounds and how learners behave in the classroom. Cultural competency, power relations, psychological safety come through strongly to me and should be labelled as such. It is not just about becoming a silent literate- it is much more.Tip 3- Provide advance notice is a relatively weak title as the point is not just about telling learners about the class ahead of time. it also includes time to reflect, time to absorb, integrate with prior learning and make connections. Saying ' I will get back to you later" may not suffice. Agree with other pointsTip 4- The authors do bring up the concept about wait time appropriately so.Tip 5- I agree with allowing a variety of contribution formats.Tip 6- While i agree with this, i wonder how distinct this is from tip 5. Maybe a more clear delineation between variety in curricular design and educational strategies might help readersTip 7- I like clear communication about who talks and when to allow introverts to talk and extroverts to listen.Tip 8- and 9- These are an excellent tips- calling things out, make the implicit explicit. Tip 11 and 12 can be classified under assessment/evaluation of impactThe paper is well written, well referenced, has great clarity, a good balance of evidence and personal experience.All health professions educators will benefit from reading this and hopefully motivated to challenge their own assumptions and biases.Overall, this paper could benefit from stronger phrases as tip titles, could have brought in principles of implicit bias, psychological safety and referring to hw these differences play out in a virtual setting.One other idea that occurred to me was that the tips could have been grouped under related categories such as below or some other:- teachers- knowing themselves, challenging their assumptions, reflecting on their biases- learners- training introverts, encouraging collaboration between introverts and extroverts- curriculum- varying contribution formats and including many styles into curriculum design- evaluation- building in assessment methods for all personality types etcI was grouping the tips into categories in my head, would have been so much easier if the authors had done this for me.

Reviewer Expertise:

NA

No decision status is available
